# The advent of preventive high-resolution structural histopathology by artificial-intelligence-powered cryogenic electron tomography

**DOI:** 10.3389/fmolb.2024.1390858

**Published:** 2024-05-29

**Authors:** Jesús G. Galaz-Montoya

**Affiliations:** Department of Bioengineering, James H. Clark Center, Stanford University, Stanford, CA, United States

**Keywords:** cryogenic electron microscopy (cryoEM), cryogenic electron tomography (cryoET), cryogenic volume electron microscopy (cryoVEM), cryogenic focused ion beam milling scanning electron microscopy (cryoFIB-SEM), cryogenic correlative light and electron microscopy (cryoCLEM), artificial intelligence (AI), machine learning (ML), structural digital and computational cellular pathology

## Abstract

Advances in cryogenic electron microscopy (cryoEM) single particle analysis have revolutionized structural biology by facilitating the *in vitro* determination of atomic- and near-atomic-resolution structures for fully hydrated macromolecular complexes exhibiting compositional and conformational heterogeneity across a wide range of sizes. Cryogenic electron tomography (cryoET) and subtomogram averaging are rapidly progressing toward delivering similar insights for macromolecular complexes *in situ*, without requiring tags or harsh biochemical purification. Furthermore, cryoET enables the visualization of cellular and tissue phenotypes directly at molecular, nanometric resolution without chemical fixation or staining artifacts. This forward-looking review covers recent developments in cryoEM/ET and related technologies such as cryogenic focused ion beam milling scanning electron microscopy and correlative light microscopy, increasingly enhanced and supported by artificial intelligence algorithms. Their potential application to emerging concepts is discussed, primarily the prospect of complementing medical histopathology analysis. Machine learning solutions are poised to address current challenges posed by “big data” in cryoET of tissues, cells, and macromolecules, offering the promise of enabling novel, quantitative insights into disease processes, which may translate into the clinic and lead to improved diagnostics and targeted therapeutics.

## 1 Introduction

Pathology is the medical specialty that studies the nature and causes of disease and has played a pivotal role in understanding, diagnosing, and treating diseases since antiquity, when early healers and philosophers began to contemplate disease etiology (van den Tweel and Taylor, 2010). Although Renaissance scholars revolutionized anatomical studies with detailed autopsies and dissections, it was botanist Matthias Schleiden, zoologist Theodor Schwann, and pathologist Rudolf Virchow who pioneered the Cell Theory in the first half of the 19th century, thereby laying down the cornerstone for modern cellular pathology (Ribatti, 2018). At the microscopic level, histology and the field of cellular pathology, also referred to as histopathology, analyze patient tissues to understand the structural and functional alterations associated with diseases (Musumeci, 2014). While histopathology and the experimental laboratory techniques it routinely relies on are already being potentiated by rapid advances in computer science and artificial intelligence (AI) (de Matos et al., 2021), unique opportunities will be forthcoming to enhance cellular pathology as machine learning (ML) methods also extend the capabilities of cryogenic electron microscopy (cryoEM) and tomography (cryoET) and related techniques, facilitating the efficient, quantitative processing of patient derived imaging datasets.

### 1.1 Techniques in structural, cellular pathology

In terms of structural characterization, histologists and histopathologists most commonly use light and/or fluorescence microscopy (LM and FM, respectively), which can be complemented by electron microscopy (EM) to gain insights at higher resolution than that allowed by the former techniques (Chen et al., 2011). Light microscopy uses visible light to visualize overall tissue architecture and cellular morphology, sufficing to detect relatively large abnormal structures such as tumors or infectious agents including bacteria and parasites. Moreover, FM utilizes fluorescent dyes or proteins to visualize specific molecules or structures within cells and tissues. These microscopy methods are commonly complemented by various molecular biology techniques to enhance specificity and detail. For instance, immunohistochemistry (IHC) is frequently employed to identify specific proteins in samples using antibodies labeled with fluorescent or chromogenic markers (Hofman and Taylor, 2013). Additionally, *in situ* hybridization (ISH) enables the detection of specific nucleic acid sequences within tissue samples by hybridizing labeled nucleic acid probes to complementary target sequences in cells (Jensen, 2014). While IHC enables pathologists to identify cell types, characterize tumors, and assess protein expression patterns, ISH allows visualizing infectious agents as well as gene expression patterns and chromosomal abnormalities to diagnose genetic disorders.

Pathologists also routinely use traditional transmission electron microscopy (TEM) and, occasionally, scanning EM (SEM) to visualize thin tissue sections (Graham and Orenstein, 2007). TEM provides detailed images of internal cellular structures, making it particularly valuable for investigating ultrastructural alterations suggestive of disease. Moreover, EM can be integrated with LM/FM through multi-scale correlative approaches that facilitate molecular assignments in high-resolution EM images by aligning them with fluorophore signals (Giepmans et al., 2005). While high-magnification EM can routinely achieve atomic resolution visualization (∼1 Å) for samples in material sciences, including those that form strong, repetitive lattices (Spence, 1999; Egerton and Watanabe, 2022), biological specimens are more susceptible to deformation from radiation damage even when stained or fixed (Luther, 2006), and are typically visualized at lower magnifications that can capture large sections of tissues, entire cells, organelles, and macromolecular complexes, yielding resolutions at nanometric scale. Furthermore, since IHC and ISH rely on fluorescent signals, histopathological observations using these methodologies are diffraction-limited to ∼200–500 nm resolution, depending on the wavelength of light used in the optical microscope.

Although traditional tissue-based evaluations of histopathology slides through direct microscopy observation, typically using single biomarkers, do not lend themselves to complex quantitative analyses, advances in staining techniques allowing for multiplex immunohistochemistry and immunofluorescence have enhanced the diagnostic accuracy and specificity of cellular pathology and will soon become routine in the clinic (Harms et al., 2023). Furthermore, the digital revolution of the last few decades has permeated every facet of medicine, and pathology is no exception. With the emergence of digital pathology (Hanna and Pantanowitz, 2019) and whole-slide imaging (Madabhushi and Lee, 2016), encompassing the digitization and computational visualization of histological slides, modern examinations are increasingly occurring virtually without being tethered to the microscope or the laboratory. This has ushered in a new era of telemedicine, efficiency, and remote collaborations in tissue-based diagnostics, and arguably represents the way of the future for the field (Pallua et al., 2020). Importantly, this digital revolution has rendered a plethora of histopathology data amenable to automated quantitative image analysis (Gurcan et al., 2009), thereby giving rise to computational pathology (Louis et al., 2014), which uses mathematical models and computational methods to extract relevant, clinically actionable information from various sources of raw data. The computational methods used include modern AI approaches (Niazi et al., 2019), such as ML leveraging deep convolutional neural networks (CNNs) (Banerji and Mitra, 2022). These applications are making their way into the clinic (van der Laak et al., 2021) and allowing for large-scale data mining to improve validation and derive population-level insights. These techniques streamline workflow efficiency by automating routine tasks, such as tissue segmentation and feature extraction, thereby augmenting pathologists’ diagnostic capabilities, speed, and accuracy.

### 1.2 Volume EM as a bridge toward high-resolution 3D histopathology

**FIGURE 1:**
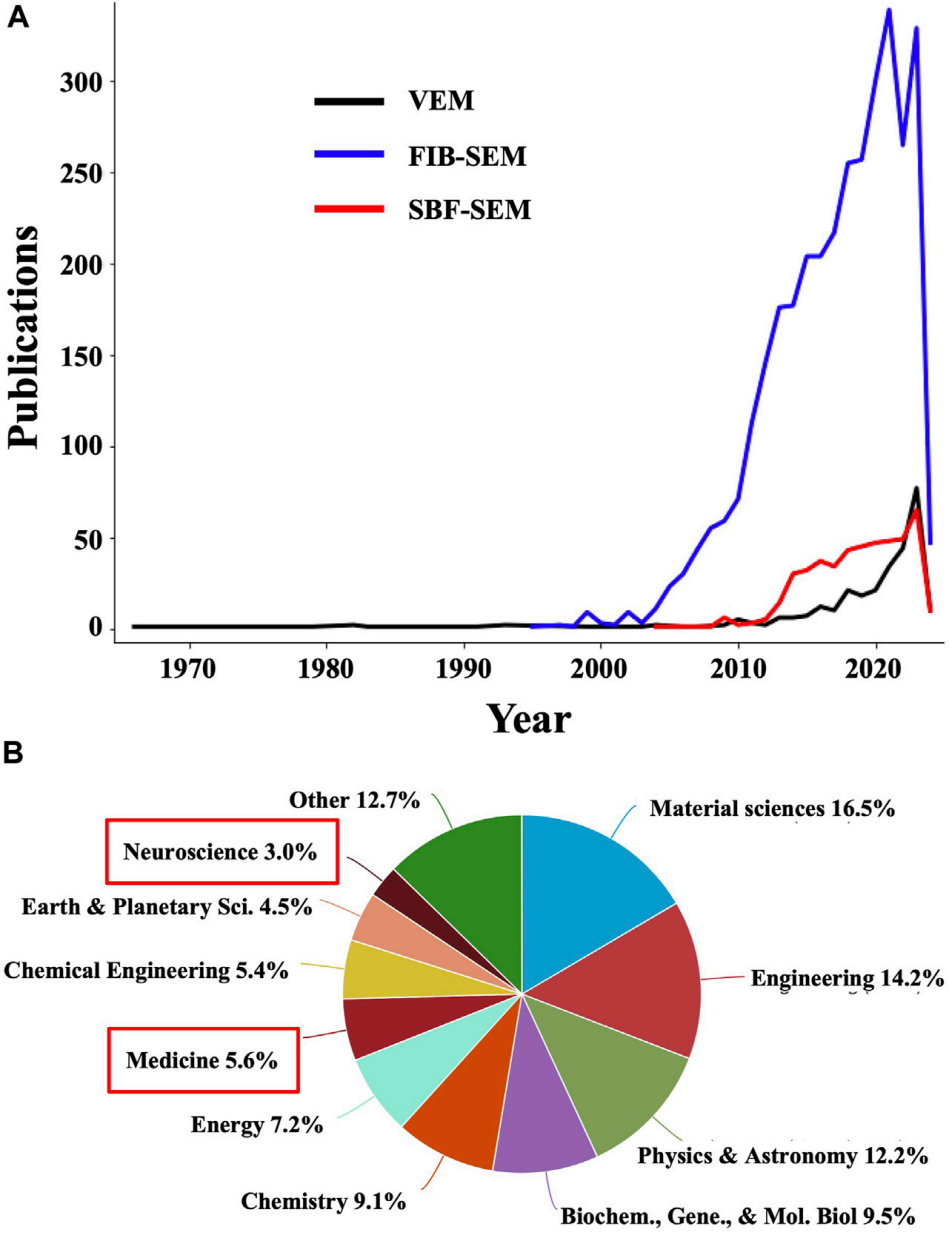
The number of publications using volume EM and related methodologies is undergoing exponential growth. **(A)** Plots showing the number of publications in Elsevier’s Scopus database containing “volume EM” OR “volume electron microscopy” OR “volumetric electron microscopy” (black line); “focused ion beam scanning electron microscopy” OR “FIB-SEM” OR “FIBSEM” OR “FIB SEM” OR “focused ion beam SEM” OR “FIB scanning electron microscopy” (blue line); and “serial block-face scanning electron microscopy” OR “serial block face scanning electron microscopy” OR “serial block face SEM” OR “serial block-face SEM” (red line). **(B)** Distribution of scientific disciplines represented in the combined publications in **(A)**. Medicine and neuroscience, highlighted with red boxes, are underrepresented and thus constitute areas of opportunity for an increased application of VEM and related techniques.

Volume electron microscopy (volume EM or VEM) refers to a group of cutting-edge imaging techniques that generate serial images of resin-embedded cellular or tissue specimens that are at least ∼1 μm thick (Peddie and Collinson, 2014). Collinson et al., 2023 highlighted this technique in a recent Nature Methods review, deeming it “a revolution in the making”. This recognition is well founded since VEM techniques are yielding unprecedented insights into cellular and subcellular architecture through large volumes of biological samples, in 3D, at the nanoscale level, in contrast to traditional EM, which typically provides two-dimensional (2D) projection images of thin samples. Indeed, while the number of publications containing VEM-related terms is still small, it’s increasing exponentially (Figure 1). Moreover, the number of publications containing terms referring to the main VEM techniques, namely, focused ion beam (FIB) scanning electron microscopy (SEM) and serial block-face (SBF) SEM, are much larger (Figure 1A). Another powerful albeit less common VEM technique is array tomography (Smith, 2018), for which publication numbers are also growing, although at a slower pace. Interestingly, when analyzing the scientific areas represented in the combined publications including terms for VEM, FIB-SEM, and SBF-SEM, “Medicine” is extremely underrepresented, accounting for merely 5.6% among the total number of publications using these techniques (Figure 1B). While cost, accessibility, and throughput remain a concern, such factors don’t seem to have impeded a more active use of these technologies in other fields. While this may be in part due to FIB milling being originally developed for room-temperature applications in material sciences, it also points to a relatively untapped trove of opportunities for biomedical imaging as VEM methods are made more accessible and efficient, especially through integration with AI-based strategies (Kievits et al., 2022), as demonstrated in a recent study analyzing hepatoblastoma patient tissues using ML for comprehensive segmentation of SBF-SEM reconstructions (de Senneville et al., 2021).

In general, VEM generates serial sections from thick biological samples, which are imaged using TEM or SEM with datasets then being computationally aligned and reconstructed into 3D representations of the sample, allowing researchers to better visualize complex spatial relationships between cellular structures and thereby understand their organization within tissues with improved accuracy. While primarily utilized in specialized research settings and academic institutions for detailed investigations of tissue ultrastructure and molecular composition, VEM methods are rapidly becoming more efficient (Kornfeld and Denk, 2018) and are thus primed to enhance clinical histopathological examinations. They are particularly valuable for studying complex biological systems such as the brain, where figuring out the 3D arrangement of neurons and synapses across the entire “brain connectome” is critical for fully understanding brain function (Ohno et al., 2015). Another target that is well-suited for VEM approaches is the 3D tumor microenvironment (Jadav et al., 2023). Furthermore, recent applications of VEM have revealed structural defects in samples from patients with primary ciliary dyskinesia, autoimmune disorders, and neurodegenerative diseases that were not detectable with traditional EM on thin sections (Peddie et al., 2022). The growing use of ML to improve FIB-SEM analyses is increasingly allowing researchers to investigate whole-organelle morphologies (Heinrich et al., 2021), synaptic connectivity (Dorkenwald et al., 2017; Santuy et al., 2020), and other cellular features at an unprecedented scale and with unparalleled levels of detail, both necessary for advancing our understanding of the mechanisms underlying pathological processes. When translated into the clinic, these striking advances will enable further improvements in the specificity, sensitivity, and thoroughness of histopathological diagnoses.

### 1.3 Cryogenic EM/ET and FIB-SEM at the dawn of high-resolution cellular pathology

Cryogenic electron microscopy (cryoEM) (Frank, 2006; Bendory et al., 2020) and tomography (cryoET; Frank, 2008; Wagner et al., 2017; Turk and Baumeister, 2020) offer unique advantages for visualizing biological specimens in 3D at even higher resolution than traditional EM technologies, without artifacts from chemical fixation and with fewer mechanical manipulations. These techniques involve flash-freezing biological samples in fully hydrated, near-native states (Dubochet et al., 1981; McDowall et al., 1983; Dubochet et al., 1988). Single-particle analysis cryoEM (SPA) is a cutting-edge technique for *in vitro* macromolecular structure determination at atomic (Nakane et al., 2020; Yip et al., 2020) and near-atomic (Hryc and Baker, 2022; Li et al., 2023) resolutions. This method relies on extensive classification and averaging of hundreds to millions of 2D projections of individual instances of macromolecules, also referred to as “single particles”, typically floating in different orientations in solution before being vitrified by plunge freezing.

Technological advances ranging from better specimen support grids (Russo and Passmore, 2014; Yu et al., 2016; Noble et al., 2018; Doerr, 2019), cryopreservation devices (Jain et al., 2012; Darrow et al., 2019), detectors (Levin et al., 2023; Peng et al., 2023), microscopes (Fréchin et al., 2023) and associated hardware such as energy filters (Grimm et al., 1997), cold field emission guns (Hamaguchi et al., 2019; Kato et al., 2019), phase plates (Danev and Baumeister, 2017), and increasingly sophisticated and automated software for data collection (Thompson et al., 2019) and processing (Punjani et al., 2017) have reduced the cost of macromolecular structure determination by cryoEM SPA (Chua et al., 2022), turning it from a niche technique into a mainstream method (de la Cruz and Eng, 2023). Although SPA is unlikely to find immediate applications in histopathology, its technological developments spill over and benefit cryoET and related methodologies, the latter being predictably much more likely to revolutionize cellular pathology in the years to come.

CryoET visualizes specimens in 3D by computationally reconstructing tomograms from a series of multiple 2D images taken from the same target area at different angles (Kremer et al., 1996). By virtue of its three-dimensional nature, cryoET is uniquely capable of revealing the structures of pleomorphic systems, from cells (Kürner and Baumeister, 2006) to amyloid filaments (Shahmoradian et al., 2013; Darrow et al., 2015; Bäuerlein et al., 2017; Guo et al., 2018; Galaz-Montoya et al., 2021; Trinkaus et al., 2021) and enveloped viruses (Obr and Schur, 2019; Li, 2022), including those captured while infecting cells (Grünewald and Cyrklaff, 2006; Quemin et al., 2020), among many others. Indeed, cryoET is particularly well suited for studying spatial relationships between macromolecular complexes and cellular organelles, as well as dynamic processes such as viral infection (Murata et al., 2017; Graham and Zhang, 2023; Hernandez-Gonzalez et al., 2023; Liu et al., 2024) and replication (Dai et al., 2013; Jin et al., 2018) cycles, including for SARS-CoV-2 (Klein et al., 2020). Of note, directly visualizing multiple types of complexes at molecular resolution has not been demonstrated even with the most advanced room-temperature VEM techniques; therefore, whether preceded by cryoVEM or not, cryoET has an unparalleled capability to reveal the intricacies of cellular ultrastructure at the highest resolutions in near-native states.

Automation of tilt series acquisition (Mastronarde, 2005) and ensuing tomographic reconstruction (Noble and Stagg, 2015; Mastronarde and Held, 2017; Zheng et al., 2022; Chaillet et al., 2023; Khavnekar et al., 2023) has helped cryoET become a routine technique for many specimens. Downstream subtomogram averaging (STA) (Galaz-Montoya and Ludtke, 2017; Förster, 2022) can yield structures at subnanometer (Schur et al., 2013) and near-atomic (Schur et al., 2016) resolutions, even for complexes within cells (Tegunov et al., 2021). While STA was first demonstrated nearly 30 years ago (Walz et al., 1997) using *ad hoc* scripts (Nicastro et al., 2006), the first semi-automated pipelines for STA started emerging only about a decade ago (Castaño-Díez et al., 2012; Hrabe et al., 2012; Galaz-Montoya et al., 2015), and have become increasingly sophisticated and efficient (Bharat and Scheres, 2016; Galaz-Montoya et al., 2016; Castaño-Díez et al., 2017; Himes and Zhang, 2018; Fernandez and Li, 2021; Zivanov et al., 2022; Burt et al., 2024), with the field rapidly taking large strides towards full automation for some specimens (Chen et al., 2019; Ni et al., 2022; Balyschew et al., 2023). Clearly, these advancements have positioned STA at the forefront of high-resolution structural biology *in situ*.

In the context of potential histopathological examination by cryoEM/ET, while plunge-freezing suffices to vitrify biochemically isolated macromolecular complexes and small cells bacterial cells and platelets (Dobro et al., 2010) and is also adequate to visualize the thin periphery of larger eukaryotic cells (Martins et al., 2021), or thin neuronal processes (Shahmoradian et al., 2014), high-pressure freezing (HPF) is required to properly vitrify multicellular organisms and tissues up to ∼200 µm in thickness (Moor, 1987; Dahl and Staehelin, 1989; Studer et al., 2001). Various strategies have been developed to thin down cryopreserved, thick biological specimens to render them amenable to cryoEM/ET imaging. For example, cryo-sectioning (Watkins, 2001; Griffith et al., 2008) uses an ultramicrotome to generate thin sections (∼50–500 nm thick) from vitrified samples; however, it’s an extremely challenging and slow-throughput technique that suffers from myriad artifacts that distort the frozen samples (Al-Amoudi et al., 2005). On the other hand, cryogenic FIB-SEM (cryoFIB-SEM) can mill thick specimens into lamellae more efficiently and presumably with fewer artifacts than cryosectioning (Marko et al., 2006). These lamellae can be rendered optimally thin (∼60–350 nm in thickness) for subsequent cryoET (Villa et al., 2013), though thicker lamellae (∼400–500 + nm) have also been analyzed to preserve more of the 3D cellular context near targets of interest (Wu et al., 2020). Of note, challenging specimens that exhibit relatively thick peripheries (∼400–600 + nm thickness) and are not easy candidates for cryoFIB-SEM milling have also been characterized by cryoET (Dudek et al., 2023). Irrespective of what method is used to thin out tissue samples, montage tomography (Peck et al., 2022) will likely play a central role in generating reconstructions at the highest possible resolution over large continuous areas.

### 1.4 Correlative light and electron microscopy for targeted, high-resolution structural histopathology

Among the most prolific techniques in structural histopathology, IHC and ISH combining EM and LM/FM are inherently correlative owing to their use of fluorescent probes to localize specific molecules in tissues. All high-resolution EM techniques commonly used in molecular biosciences to examine targets in cells described in prior sections can also be combined with LM/FM through correlative light and electron microscopy (CLEM) methods, including VEM techniques (Bushby et al., 2012). For example, a recent study applied correlative light and VEM to visualize morphological changes in the developing brain (Hayashi et al., 2023). While these methods may be underrepresented in the clinic owing to technical limitations and cost, they are primed to revolutionize the field of structural pathology.

CryoCLEM approaches (Lucić et al., 2007; Sartori et al., 2007; Plitzko et al., 2009; Hampton et al., 2017), including those involving cryoVEM methods (Vidavsky et al., 2016) such as cryoSBF-SEM (Hoffman et al., 2020) and cryoFIB-SEM (Gorelick et al., 2019), are not yet routine but constitute an actively developing field given their capacity to highlight molecular identities during ultrastructural examination of cells and tissues without chemical fixation and staining artifacts (Bharat and Kukulski, 2019). Increasing efforts to automate these techniques will help to make them routine for many specimens in the near future (Klumpe et al., 2021; Yang et al., 2021; Weiner et al., 2022), including bacteria and other pathogens (Liedtke et al., 2022), particularly as ML strategies are incorporated into many steps of the workflows (Seifert et al., 2020). Recent implementations have demonstrated cryoCLEM of entire cells using cryoSBF-SEM followed by cryoFIB-SEM lamellae milling and cryoET of targeted areas of interest (Wu et al., 2020), as well as for tissues using the ‘lift-out’ technique (Schaffer et al., 2019; Kuba et al., 2021) and entire organisms using ‘serial lift-out’ (Schiøtz et al., 2023). Of particular relevance to histopathology evaluations at high resolution, correlative strategies have been demonstrated even for complex montage cryoET (Yang et al., 2023), which powerfully allows researchers to stitch multiple tomograms together, thereby delivering 3D views over large, continuous areas of the imaged specimen. Exciting developments that combine cryogenic super-resolution LM and FM with cryoEM methods (Chang et al., 2014; Wolff et al., 2016; Dahlberg and Moerner, 2021), for which novel supports (Last et al., 2023) are being proposed to reduce problems with specimen heating (Dahlberg et al., 2022), will usher a new era of correlative studies with unprecedented specificity and resolution. Given cryoCLEM’s unique ability to uncover rare events in cells and tissues (Kukulski et al., 2011; Ader and Kukulski, 2017), several of the techniques falling under this umbrella term will predictably find applications in the advancement of personalized medicine through high-resolution structural cellular pathology and histopathology.

## 2 Challenges and limitations in traditional and cryogenic microscopy techniques for structural studies of macromolecules, cells, and tissues

Despite being powerful techniques to examine cells and organelles, as well as the broad localization of macromolecules within them, the resolution of standard LM/FM setups is diffraction limited (Masters, 2020), providing spatial insights at up to ∼170–250 nm resolution laterally and at ∼2–3x lower resolution axially (Valli et al., 2021), which does not allow to discern molecular details. This may preclude detection of finer changes in the structure and distribution of subcellular components, as well as of conformational changes in macromolecular complexes, which may be reflective of early-stage or mild disease phenotypes. Furthermore, in histopathology applications, the use of fluorescent probes typically requires their conjugation with antibodies as well as cell fixation and permeabilization, which, depending on the system under investigation, can induce artifacts that alter the distribution and structure of subcellular components (Yoshida et al., 2023). In general, tagging endogenous molecules with fluorescent probes or overexpressing fusion constructs can be toxic to cells and/or change the localization, distribution, structure, and function of the labeled targets (Jensen, 2012). While tissue fixation with aldehydes such as formaldehyde or glutaraldehyde is a critical step in sample preparation in traditional EM to stabilize cellular structures and prevent their degradation, it can also add to a gamut of histopathological artifacts due to chemical cross-linking and tissue shrinkage (Taqi et al., 2018). Moreover, different fixatives and other specimen preparation steps like embedding, sectioning, and heavy metal staining may differentially preserve, alter, or obscure cellular components, leading to selective or anisotropic distortions that may preclude visualizing the native morphology and composition of tissues. In addition to these caveats during specimen preparation, the next frontier in microscopy of fixed specimens, namely, VEM techniques such as SBF-SEM and FIB-SEM, remain niche, slow-throughput, and costly (Guérin et al., 2019); however, increasing automation and the incorporation of AI/ML-based technologies are rapidly making them more efficient (Peddie et al., 2022; Collinson et al., 2023).

In the cryogenic domain, while SPA can yield functional insights at extremely high resolution and has found applications in drug discovery, development, and design (Subramaniam et al., 2016; Renaud et al., 2018), this technique only works *in vitro*, typically requiring harsh biochemical purification of the complexes of interest. Interestingly, SPA has recently been demonstrated for complexes in cell lysates, which is arguably closer to their native context than visualizing them in highly purified solutions (Yi et al., 2019). Nonetheless, by virtue of imaging thin layers of frozen specimens in solution or lysates, macromolecules are exposed to denaturing forces at the air-water interface during SPA experiments (Glaeser and Han, 2017). Because of these and other issues, SPA might prove less applicable to histopathological diagnostics in the future than cryoVEM methodologies, including cryoSBF-SEM and cryoFIB-SEM, with or without subsequent cryoET and STA.

Although methodologies and protocols are continuously improving, the technical difficulty and gross artifacts of cryo-sectioning have precluded it from becoming more widely applicable and routine even in basic science laboratories (Titze and Genoud, 2016). On the other hand, a big limitation of cryoFIB-SEM milling in producing lamella for imaging at high resolution with cryoET is that this hybrid method currently only allows for imaging relatively small, localized regions within cells and tissues, with most of the frozen material being burned off by the milling process. Furthermore, while cryoFIB-SEM artifacts are commonly regarded as relatively mild compared to overt cryo-sectioning crevasses, damage from the ion beam is not constrained to the surface of lamellae. On the contrary, ion beam damage to the specimen has been demonstrated to propagate through tens of nanometers into milled lamellae (Giannuzzi and Stevie, 1999; Volkert and Minor, 2007), from both surfaces, with the extent of damage depending on the sample, the nature of the ion beam (*e.g.,* using gallium vs. argon ions) (Berger et al., 2023; Lucas and Grigorieff, 2023), and the accelerating voltage used to mill (Mayer et al., 2007).

Localizing regions of interest to mill lamellae has proven to be another significant challenge, particularly for multicellular and tissue specimens (Navarro, 2022). While correlative techniques can aid in such a task, particularly developments incorporating super-resolution techniques, the limited number of fluorescent probes amenable to imaging at cryogenic temperatures limits the wider applicability of these techniques (Dahlberg et al., 2018). In addition, the risks of contamination and devitrification are high due to multiple specimen transfers between microscopes and other pieces of equipment for most setups. Even when the targets of interest can be localized within frozen cells or tissues on cryoEM grids, the difficulty in adding high-contrast fiducial markers after cryoFIB-SEM lamella milling complicates the downstream task of cryoET reconstruction, depending on lamella thickness and the contrast of features in the sample (Harapin et al., 2015). Furthermore, cryoEM support grids and electron microscope specimen stages used in the life sciences are not designed to allow for full-range tilting. Even if full-range tilting were possible, the slab-geometry of frozen-hydrated specimens and milled lamellae increases the mean free path of electrons through the specimen as the tilt angle increases, yielding high-tilt images of limited value, and eventually occluding the electron beam completely at high tilt angles (Galaz-Montoya and Ludtke, 2017). Indeed, historically one of the greatest limitations in cryoET stems from the tilt range for productive data collection being limited to ± 60° for most specimens, which gives rise to the so-called “missing wedge” and its associated artifacts, such as anisotropic resolution in the reconstructed tomograms (Radermacher, 1988). These artifacts negatively affect downstream processes such as feature identification, segmentation, and quantitative analyses.

Although not a direct experimental limitation, the raw frame images and downstream files generated during data collection and processing by cryoEM/ET comprise many terabytes of data for each specimen examined. This is a consequence of modern direct electron detectors (Milazzo et al., 2005) enabling the acquisition of multiple images or “frames” of the same specimen area at a rapid rate, with low electron doses, which can then be aligned to derive averaged images with much higher contrast and signal to noise ratio (Veesler et al., 2013). Furthermore, these detectors are becoming faster and larger (McMullan et al., 2016), guaranteeing a more rapid rate of dataset growth for cryoEM and related techniques which, on top of everything, are becoming increasingly popular (Callaway, 2020). By now, it is widely recognized that cryoEM and cryoET are “big data” methods (Baldwin et al., 2018) that pose myriad associated challenges in data storage, transfer, processing, and management in general, requiring advanced and costly computational resources (Poger et al., 2023). A related challenge in the field is that many academic software applications are developed for *ad hoc* purposes by non-professional programmers and thus can often be suboptimally designed as well as poorly distributed, documented, and maintained, particularly in fields that are not computationally focused. While many impressive tools have been developed by the academic community for analysis of datasets generated by cryoEM/ET and related techniques, their practical use and wide adoption are often not without challenges due to limited dissemination, scant quality metrics and benchmarking studies, particularly for cryoET tomograms in the absence of STA, lacking standardization of data formats, as well as complexities in software installation, usage, maintenance, compatibility with heterogeneous hardware systems, and interoperability across operating systems and complementary software upstream or downstream in the data processing pipeline.

## 3 Current AI applications in cryoEM as a routine technique and cryoET at the frontier of high-resolution cellular biology

**FIGURE 2:**
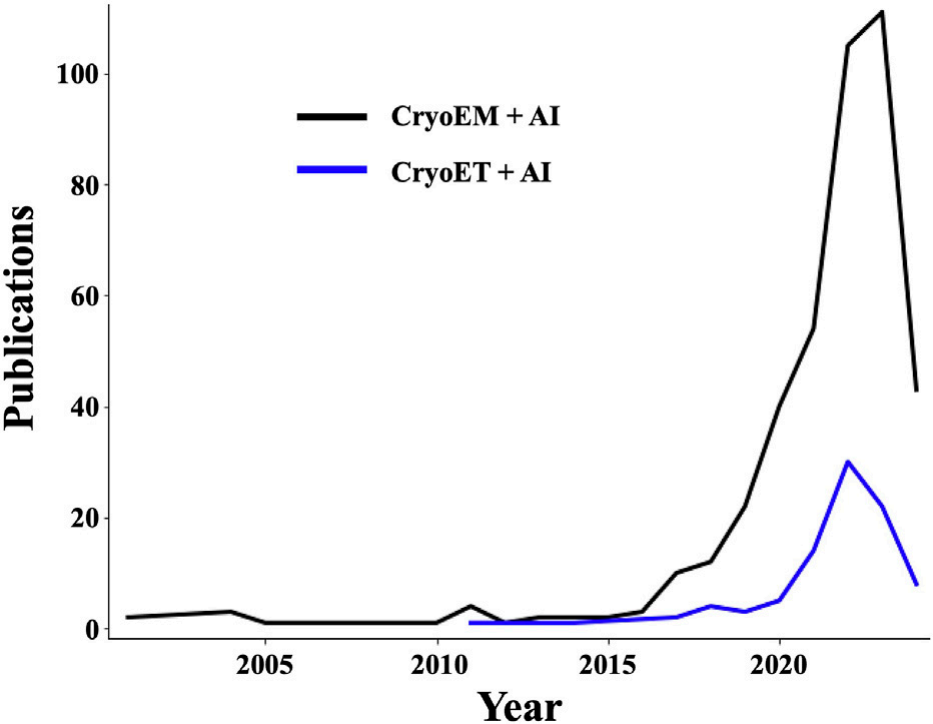
The number of publications showcasing the use of AI in cryoEM/ET applications is undergoing exponential growth. Plots showing a growing trend in the number of publications in Elsevier’s Scopus database containing (“cryoem” OR “cryo-em” OR “cryogenic electron microscopy” OR “cryo electron microscopy”) terms (black curve), or (“cryogenic electron tomography” OR “Subtomogram averaging” OR “cryo-et” OR “cryoet” OR “cryo electron tomography”) terms (blue curve), in conjunction with (“deep learning” OR “artificial intelligence” OR “machine learning” OR “neural networks”) terms.

Recent ML and AI applications have been increasingly contributing to the rapid rise of cryoEM since the turn of the millennium but most prominently over the last 6 years (Figure 2), enabling efficient, unbiased workflows that can reconstruct SPA structures from cell extracts without knowing the identities or relative abundance of their biochemical components *a priori* (Skalidis et al., 2022). These types of feats have been possible and will become increasingly common thanks to AI-assisted acceleration and improvement in various workflow steps. These include specimen screening before data collection (Bouvette et al., 2022; Cheng et al., 2023), micrograph denoising (Tegunov and Cramer, 2019; Bepler et al., 2020), structure reconstruction (Giri et al., 2023), and postprocessing (Sanchez-Garcia et al., 2021), with recent efforts having been particularly concentrated on particle picking (Wang et al., 2016; Zhu et al., 2017; Sanchez-Garcia et al., 2018; George et al., 2021) and model building (He et al., 2022; DiIorio and Kulczyk, 2023; Giri et al., 2023). Machine learning approaches have sped up cryoEM SPA structure determination to the point that, for many specimens, including those exhibiting compositional and conformational heterogeneity (Zhong et al., 2021), multiple structures at near-atomic resolution can be derived in a few days, sometimes even from a single imaging session and processing the corresponding data using a single workstation equipped with GPU acceleration (Kimanius et al., 2016). Of note, the methodological similarities between cryoEM SPA and cryoET STA facilitate a mutually beneficial transfer of technologies across these methodologies.

For cryoET, the first AI applications emerged about a decade ago but have only taken off over the last 4 years (Figure 2) coming mostly from a few research groups (Zeng et al., 2021b). The main foci of attention have been the detection (Moebel et al., 2021; Lamm et al., 2022), classification (Moebel and Kervrann, 2022), and alignment (Zeng et al., 2021a) of macromolecular complexes in tomograms as well as feature annotation (Chen et al., 2017; Genthe et al., 2023). Automated and semi-automated solutions to the related problems posed by particle picking and feature annotation are of particular significance since thoroughly accomplishing these tasks manually for large cryoET datasets is outright impossible at realistic timescales, even with efficiency-driven strategies (Danita et al., 2022), and the process is subject to high levels of inconsistency and bias (Hecksel et al., 2016). Some of the most exciting AI-powered developments in cryoET are forefront methods that use CNNs to restore the missing wedge (Liu et al., 2022; Zhang et al., 2023). Another impressive application is the “deep iterative subtomogram clustering approach” (DISCA), a fully automated, label-free, and template-free pattern mining algorithm that uses CNNs to extract and cluster rotationally and translationally invariant features as subtomogram classes (Zeng et al., 2023), which can then be aligned and averaged as a post-preprocessing step.

## 4 Discussion: opportunities to further potentiate large-scale cryoET analyses of cells and tissues with AI towards enabling preventive, high-resolution structural histopathology diagnosis

From ancient observations to modern innovations, the trajectory of pathology reflects humanity’s enduring quest to unravel the mysteries of disease. Anatomical, cellular, molecular, and digital pathology represent complementary chapters in this ever-evolving quest, which is well poised to incorporate insights from the leading edge of high-resolution structural biology, namely, cryoEM/ET and related methodologies (Supplementary Figure S1). The synergistic integration of the flourishing vanguard technologies reviewed here will help propel humanity toward a future of improved diagnostics and targeted therapies at the dawn of personalized medicine.

Even though cryoEM SPA is regarded as less likely to directly apply to histopathological diagnostics any time soon compared to cryoET, cryoSBF-SEM, and cryoFIB-SEM, because it requires harsh biochemical purification or cell lysis, it’s conceivable that future technological breakthroughs may allow to cost-effectively determine structures of macromolecules purified from patient tissues to derive clinically relevant information that could complement clinical proteomics (Petricoin et al., 2004; Mani et al., 2022) in the delivery of personalized medicine. Indeed, several structures of polymorphic amyloidogenic tau filaments have been resolved to high resolution by cryoEM SPA, including from samples purified from patient tissues (Scheres et al., 2020). Interestingly, a recent and exciting study that reported a newly discovered amyloidogenic motif, Polymorphic Amyloid Motif of Repeat 4 (PAM4), replicated several of these structures *in vitro* by cryoEM SPA of a synthetic PAM4 peptide (Louros et al., 2024). However, in spite of the challenges comprehensively reviewed here, cryoET and STA downstream of cryoVEM and cryoFIB-SEM lamellae milling are likely to play a more central role in achieving the promise of high-resolution structural histopathology diagnostics directly from patient tissues and cells or patient-derived models in a near-native state and context, such as organoids and iPSCs.

While reproducible specimen preparation and grid screening have become highly efficient and routine for many cryoEM SPA targets, these steps remain a more significant initial bottleneck in cryoET, particularly for multicellular organisms and tissues. Once specimens have been prepared, automatically identifying good areas for tilt series collection is one of the challenges to overcome in the near future that will resolve a significant bottleneck, as recently accomplished for single particle cryoEM with the help of AI algorithms (Yokoyama et al., 2020). After specimen preparation and grid screening, successful automated acquisition of tilt series is comparatively fast and routine, and the possibility of boosting this step has been recently demonstrated by emerging methods such as continuous and fast incremental tomography (Chreifi et al., 2019). Approaches leveraging AI/ML could conceivably help to automatically “prune” low-quality images during or right after data collection by learning to identify and exclude images with anomalous defocus, large stage drift, poor contrast, radiation damage, excessive contamination, reflections from non-vitreous “bad ice”, and/or specimen charging causing uncorrectable image blurriness. This would reduce the amount of parasitic data downstream, precluding corresponding storage and processing overhead burdens. Additionally, strategies that sample milled lamellae more efficiently—like parallel (Eisenstein et al., 2023) and montage (Peck et al., 2022) tomography—are already improving data collection for cryoFIB-SEM milled samples. Of note, the latter allows visualizing large continuous areas of the specimen by stitching together multiple sequential reconstructions from adjacent areas, constituting an important stepping stone towards building high-resolution molecular atlases over large regions in cells and tissues. Importantly, montage cryoET has recently been demonstrated in conjunction with correlative techniques (Yang et al., 2023). A relatively neglected albeit useful strategy is the addition of gold fiducial markers to lamellae after cryoFIB-SEM milling (Harapin et al., 2015). While many software packages can now successfully align tilt series of thin specimens without fiducials, quality is often compromised as most specimens yield low-contrast images, particularly ones near the thickness limits for cryoET (∼500–600 nm), precluding optimal tilt series alignment and tomographic reconstruction. Continued development of phase plates (Danev and Baumeister, 2017), and AI-driven denoising methods (Palovcak et al., 2020) to enhance image contrast will predictably increase the number of datasets that can be optimally reconstructed in the absence of gold fiducial markers.

CryoEM/ET and related imaging modalities have been recognized as “big data” methods that meet the definition criteria of rapidly producing large amounts of varied data (Baldwin et al., 2018; Poger et al., 2023). As such, data management, processing, and analysis have become larger bottlenecks than data collection. While the quest for efficient data representations in cryoEM/ET is not new, and has been addressed in a few studies (Fluty and Ludtke, 2022), increasing the efficiency of storage and transfer of high-dimensionality data is still an area of substantial opportunity. This challenge could benefit from the development of unsupervised ML techniques that integrate statistical methods like Principal Component Analysis and autoencoders to achieve effective data compression without significant loss of information. Furthermore, simulations and supervised machine learning approaches training on existing data could be leveraged to create predictive models that facilitate experimental parameter optimization, thereby saving time and computational resources. Recent algorithms have demonstrated rapid, automated, even on-the-fly tomographic reconstruction (Zheng et al., 2022), including accurate and detailed determination of the contrast transfer function and astigmatism for tilted specimens (Mastronarde, 2024), as well as missing wedge restoration by CTF deconvolution (Croxford et al., 2021) and other methods. Indeed, multiple increasingly automated pipelines have emerged to expedite cryoET workflows (Morado et al., 2016; Böhning and Bharat, 2021), including subtomogram averaging, tomographic annotation, and software interoperability (Jiménez de la Morena et al., 2022). Despite such exciting and fast-paced progress, the use of ML to automate tasks in cryoET and related techniques is in its infancy, begging for increased and closer collaboration between biological scientists and microscopists with computer scientists, as well as between academia and the healthcare, medical, pharmaceutical, and biotech industries (Garousi et al., 2016). Indeed, given how computationally and data-intensive cryoEM/ET analyses are, there is a great need for efficient, cross-platform, easy-to-install, well-maintained, well-documented, and user-friendly software for data processing and analysis. Moreover, the availability of AI-based tools for coding and software development (Wu T. et al., 2023; Moradi Dakhel et al., 2023) could help in the attainment of such a goal. Moving into the future, increased file format standardization, improved software documentation and maintenance, and adoption of best practices guided by extensive benchmarking (Turoňová et al., 2020) will contribute to reducing post-data collection bottlenecks.

In terms of alleviating missing wedge distortions, redesigned specimen supports, as well as holders and stages for electron microscopes to enable in-plane rotation of grids and/or full-range tilting as in might prove to be particularly rewarding. A proof of concept for this, namely, the development and usage of carbon nanopipettes as cylindrical specimen supports, was published a decade ago (Palmer and Löwe, 2014) but has failed to gain traction. Furthermore, the unique potential of using three orthogonal projections to minimize mathematical ambiguity in the orientation and conformation of single particles was qualitatively illustrated also a decade ago (Galaz-Montoya, 2014), a concept that could be extended to collecting tri-orthogonal images of cells and tissues, thereby experimentally precluding the missing wedge problem altogether. Alternatively, emerging AI algorithms in computer vision such as vision transformers (Khan et al., 2022), foundation models (Ma and Wang, 2023), and coordinate networks (Tancik et al., 2020), which outperform CNNs in many tasks, might help to produce the next-generation of AI-powered solutions for cryoEM/ET and related technologies, particularly in improving missing wedge restoration as well as feature segmentation, classification, and downstream quantitative analyses. Coordinate networks have indeed recently been demonstrated to outperform the leading CNNs implementation for missing wedge restoration (Liu et al., 2022) in terms of shape fidelity and reconstruction efficiency (Van Veen et al., 2024), albeit this was only through *in silico* experiments and replication of these results on cryoET data is pending.

The rise of cryoCLEM methods has alleviated the issue of specimen localization in cryoFIB-SEM milled specimens to some extent but these techniques still suffer from poor axial resolution. On the other hand, the development of super-resolution light microscopy techniques such as 4pi microscopy, stochastic optical reconstruction microscopy (STORM), stimulated emission depletion (STED) microscopy, and photoactivated localization microscopy (PALM) have bridged the gap between LM/FM and EM (Henriques et al., 2011), allowing microscopists to visualize biological systems at resolutions between ∼20 and 100 nm. Furthermore, these techniques can be correlated with EM to gain increased molecular details (Timmermans and Otto, 2015). For example, a recent seminal study used a combination of STED, SBF-SEM, TEM, and STA with biophysical techniques to discover unique structural features never described before in Lewy pathology, directly in Parkinson disease patient post-mortem brain tissues (Shahmoradian et al., 2019). Other promising studies have recently demonstrated the use of cryoCLEM and cryoET to examine the structures of amyloid plaques *in situ* directly in cryogenic ultramicrotomy slices from rodent brains (Leistner et al., 2023), as well as the use of xenon plasma-based cryoFIB milling followed by cryoET to examine cellular structures in post-mortem human brain tissues (Creekmore et al., 2024). An exciting preprint showcasing a tour-de-force combining extensive cryoCLEM, cryosectioning, cryoFIB-SEM lift-out, and cryoET examined the structures of fibrillar amyloid-β and tau inclusions directly in post-mortem brain tissues from Alzheimer disease patients and control donors (Gilbert et al., 2023).

On the specimen front, patient-derived organoids are emerging as extremely useful model systems (Ooft et al., 2019) that may guide the development of improved, targeted treatments (Chen et al., 2021). These and patient-derived induced pluripotent stem cells (iPSCs) (Malik and Rao, 2013) represent huge steps toward realizing the dream of minimally invasive diagnostics and will play a prominent role in structural histopathology given the demonstrated feasibility of imaging them with cryoVEM techniques and cryoET. For example, a recent cryoET study examining neurons differentiated from Huntington disease (HD) patient-derived iPSCs identified unique structural phenotypes in neurite organelles, likely corresponding to early disease stages, and validated their rescue upon knockdown of a candidate therapeutic target (Wu G.-H. et al., 2023). Further studies of these systems leveraging cryoFIB-SEM lamella milling could help confirm whether the observed phenotypes are also reflected in altered structures through the much thicker neuronal cell body or even in post-mortem, patient-derived brain tissues. Along those lines, automated fiducialless alignment strategies have remedied the problem of lacking gold fiducials in tilt series from cryoFIB-SEM lamellae for favorable cases in which the specimen has been thinned out enough and high-contrast features are present, well distributed, and trackable within the field of view throughout the tilt series, including at high-tilt angles. Ongoing denoising and alignment strategies powered by AI may facilitate the accurate reconstruction of tomograms from tilt series of lamellae on the thicker end of the spectrum lacking fiducials.

The emergence of super-resolution cryoCLEM methods offers the promise to yield a plethora of exciting observations from samples in fully hydrated, near-native conditions. There’s great diagnostic potential in such types of findings if efficient protocols and pipelines can be developed to reduce turnaround time and cost. The marked increase in interest in cryoEM/ET, cryoVEM, and related techniques, as well as their rapid democratization (Stuart et al., 2016; Serbynovskyi et al., 2024), as evidenced by the opening of national service centers for macromolecular structure determination (Eng et al., 2019), training (Eng et al., 2023), and cryoET specimen preparation (Larson et al., 2022) in the USA constitute foreshadowings of the promising potential of these technologies to advance biomedicine, particularly drug design and the nascent field of high-resolution, near-native-state cellular structural pathology and histopathology championed in this review.

## 5 Conclusion

Although cryoEM/ET and super-resolution light microscopy techniques typically suffer from lower throughput compared to conventional microscopy methods, as the medical field embraces what Peter Attia calls the “Medicine 3.0” paradigm (Attia, 2023), turnover time may become less critical to many clinical diagnoses given the preventive nature of this model that will usher humanity away from “sick-care” toward genuine “healthcare”. Medicine 3.0 focuses on proactive, early interventions, thereby reducing the urgency of speedy diagnostic assays, in contrast to the demands of currently prevalent sick-care systems (Friebe, 2022) that are primarily reactive to acute pathologies at advanced stages. Increased and improved diagnostic capabilities will enable healthcare to transition from disease detection based on gross distortions in the biochemical signatures and the structural architectures of organs, tissues, and cells, to disease prevention based on high-resolution visualization of early abnormal alterations. This review is somewhat of a cryogenic analog to a recent call to increasingly use 3D EM techniques to quantitatively examine organelles as a proxy for disease monitoring (Neikirk et al., 2023). The application of iteratively improved ML models to mine growing, multi-scale, “big data” cryoVEM and cryoET datasets of patient tissues and cells should enable increasingly advanced and accurate image restoration, pattern identification, and feature classification at nanometric resolution, thereby affording greater statistical confidence to the interpretation of subcellular and macromolecular structures corresponding to early disease phenotypes, essential to realizing the possibility of personalized, preventive structural pathology.

## Supplementary material

SUPPLEMENTARY FIGURE S1 Schematic diagram showing a high-level overview of alternative coarse-grained workflows for imaging biological samples in microscopy studies involving cryogenic electron tomography (cryoET), focused ion beam (FIB) scanning electron microscopy (SEM), and/or correlative light and electron microscopy (CLEM). Three prominent fixation methods are outlined in purple boxes near the top, with colored lines representing the shortest experimental pathways for the four specific techniques highlighted in this review. These efficient paths are mostly applicable to thin specimens such as small unicellular organisms or the periphery of single cells while the dashed lines indicate more complex, alternative paths that can be followed depending on specific experimental requirements and objectives, typically suitable for thicker cells, multicellular organisms, and tissues. All paths lead down to orange boxes that highlight the reconstruction outcomes for final visualization of the imaged specimens (namely a tomographic reconstruction or a volume made of adjacent FIB-SEM slices). Note: The diagram and its connections may not be exhaustive and each pathway could be broken down into many more fine-grained experimental steps that are out of scope for this review.
