# Supplementary material for: The advent of preventive high-resolution structural histopathology by artificial-intelligence-powered cryogenic electron tomography
Source: Front Mol Biosci. 2024 May 29;11:1390858. doi: 10.3389/fmolb.2024.1390858 (PMC11167099; doi:10.3389/fmolb.2024.1390858)
Supplement: Supplementary file 1 [file Image1.pdf]

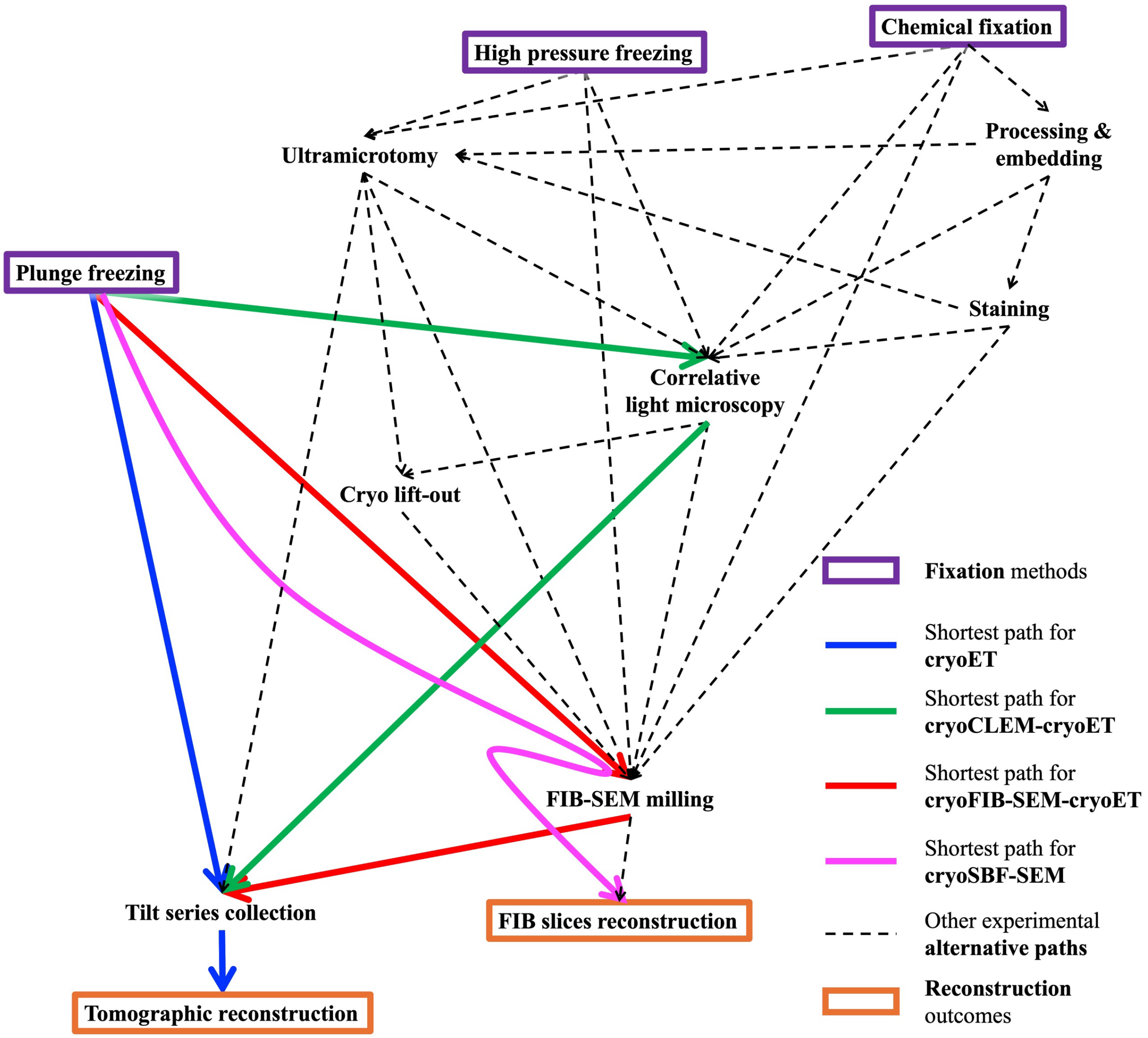

Supplementary Figure 1. Schematic diagram showing a high-level overview of alternative coarse-grained workflows for imaging biological samples in microscopy studies involving cryogenic electron tomography (cryoET), focused ion beam (FIB) scanning electron microscopy (SEM), and/or correlative light and electron microscopy (CLEM). Three prominent fixation methods are outlined in purple boxes near the top, with colored lines representing the shortest experimental pathways for the four specific techniques highlighted in this review. These efficient paths are mostly applicable to thin specimens such as small unicellular organisms or the periphery of single cells while the dashed lines indicate more complex, alternative paths that can be followed depending on specific experimental requirements and objectives, typically suitable for thicker cells, multicellular organisms, and tissues. All paths lead down to orange boxes that highlight the reconstruction outcomes for final visualization of the imaged specimens (namely a tomographic reconstruction or a volume made of adjacent FIB-SEM slices). Note: The diagram and its connections may not be exhaustive and each pathway could be broken down into many more fine-grained experimental steps that are out of scope for this review.
